# Injectable hydrogel nanoarchitectonics with near-infrared controlled drug delivery for in situ photothermal/endocrine synergistic endometriosis therapy

**DOI:** 10.1186/s40824-023-00442-2

**Published:** 2023-10-07

**Authors:** Wei Tian, Chenyu Wang, Ran Chu, Haiyan Ge, Xiao Sun, Mingjiang Li

**Affiliations:** 1 Department of Gynecology, Shandong Provincial Hospital Affiliated to Shandong First Medical University, Jinan, Shandong China; 2 School of Chemistry and Pharmaceutical Engineering, Medical Science and Technology Innovation Center, Shandong First Medical University and Shandong Academy of Medical Sciences, Jinan, China

**Keywords:** Injectable hydrogels, Controlled drug delivery, Photothermal therapy, Endocrine therapy, Endometriosis, Letrozole

## Abstract

**Background:**

Endometriosis is a common gynecological disease in women of childbearing age. Commonly used treatment methods, such as endocrine and surgical therapies, display poor therapeutic effects with a high relapse probability. Thus, novel treatments for endometriosis are required.

**Methods:**

In our study, polydopamine (PDA), letrozole (LTZ), and agarose (AG) hydrogels were combined to construct an injectable hydrogel with near-infrared controlled drug delivery named LTZ-PDA@AG hydrogel for endometriosis treatment. The release of letrozole can be accurately controlled by the near-infrared light intensity, exposure duration, polydopamine concentration, and hydrogel composition. Meanwhile, we isolated endometrial stromal cells from endometrium in patients with endometriosis, and constructed the rats’ model of endometriosis to verify the biological effects of LTZ-PDA@AG hydrogel.

**Results:**

Owing to the sufficiently deep penetration of near-infrared light, the LTZ-PDA@AG hydrogel displayed a high temperature increase for efficient photothermal therapy. In addition, high local temperatures can further enhance the diffusion and penetration of letrozole, thereby achieving excellent therapeutic effect in vivo. Importantly, the in vivo and vitro test demonstrated the capacity of the nanocomposite hydrogel for endocrine-photothermal synergistic therapy and the biocompatibility.

**Conclusion:**

Our work proposes a novel concept for precision endometriosis therapy by photothermal-enhanced endocrine therapy for endometriosis, which is proposed for the first time for the treatment of endometriosis and demonstrates excellent potential for further clinical translation.

**Trial registration:**

Not applicable.

**Graphical Abstract:**

LTZ-PDA@AG hydrogels were synthesized and displayed a high temperature increase for efficient photothermal therapy under NIR. The present study shows the capacity of the nanocomposite hydrogel for endocrine-photothermal synergistic therapy and the biocompatibility.

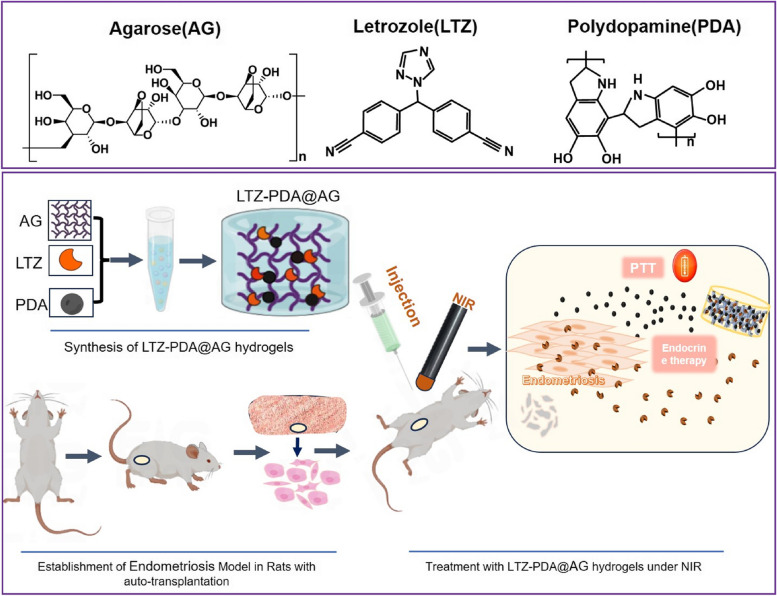

**Supplementary Information:**

The online version contains supplementary material available at 10.1186/s40824-023-00442-2.

## Highlights

An injectable agarose hydrogel composed of polydopamine and letrozole (AHPL) for synergistic photothermal and endocrine therapies of endometriosis.

The release of letrozole can be accurately controlled by the near-infrared light intensity, exposure duration, polydopamine concentration, and hydrogel composition.

AHPL displayed a high temperature increase for efficient photothermal therapy.

AHPL has high biocompatibility, and can be completely degraded in vivo.

**Supplementary Information:**

The online version contains supplementary material available at 10.1186/s40824-023-00442-2.

## Introduction

Endometriosis is defined as the presence of endometrial glands and stroma outside the uterine cavity, mainly in, but not limited to, the pelvic cavity. It is the most common cause of chronic pelvic pain [1]. Endometriosis usually causes chronic pelvic pain, infertility, menstrual irregularity, dyspareunia, and impaired quality of life [2]. About 5–10% of women of reproductive age have this condition, and the prevalence increases to 20–50% in infertile women [3]. The pathophysiology and etiology of endometriosis are yet to be identified, and the exact pathophysiology of this disease remains uncertain; however, it is well known that endometriosis is an estrogen-dependent condition, while the symptoms are cyclic parallel to the menstrual cycle and resolve after menopause [4]. Several optional etiological theories have been proposed. Thus, novel treatments for endometriosis are required.

Estradiol 17b (or estrogen) is the main biochemical component involved in the growth of endometriotic implants. Aromatase is a key enzyme in the pathogenesis of endometriosis that converts precursor steroids into estradiol and estrogen and promotes the proliferation of endometrial tissue. Additionally, aromatase plays an important role in the inflammatory response that causes the disease to progress [5]. Thus, the inhibition of aromatase could be an effective way to treat and control endometrial tissue proliferation. LTZ is well known as the most effective non-steroidal third-generation aromatase inhibitor drug that prevents excess estrogen biosynthesis within the body [6–8]. In recent years, LTZ has been used as a drug against estrogen receptor-positive breast cancer and treatment of endometriosis [9–13]. LTZ has been demonstrated to suppress estrogen production both locally and systemically, and significantly reduce symptoms [13–15]. However, the adverse effects of LTZ limits its long-term use. This necessitates urgently developing better drugs and methods of drug administration.

Photo responsive hydrogels are ideal platforms for controlled drug release because of their minimal invasiveness and potential for controlled release [16]. In general, emerging two-dimensional nanomaterials such as MoS_2_ [17, 18] and WS_2_ [19–21] in transition metal disulfides (TMDs), are commonly used as photothermal energy exchangers (PTAs) because of their near-infrared (NIR) response characteristics and ease of use [22, 23]. However, most of them are based on metals, and their biological safety cannot be guaranteed, with several limitations, such as weak photothermal conversion efficiency and relatively low biodegradability, making them difficult to metabolize in the human body [24, 25]. These are practical challenges in their clinical applications [26, 27]. Based on the research of Qiu et al., a hydrogel with agarose (AG) as the photothermal controlled-release carrier was explored, and an intelligent drug-release platform with NIR response was constructed by regulating the proportion of various components in the hydrogel [28]. AG carriers are widely recognized as safe materials approved by the US Food and Drug Administration (FDA), and are simple, efficient, and biodegradable biomaterials. At present, responsive hydrogel systems have been used in wound healing [29–31], tumor-targeted drug delivery [32–34], tumor post-operative treatment [16, 35–37], bone-tissue repair, and other disease treatment fields [38–41]. However, such systems have no precedent in gynecological diseases, especially endometriosis; therefore, designing a controllable responsive hydrogel system for the treating endometriosis has great scope for application.

**Scheme 1:**
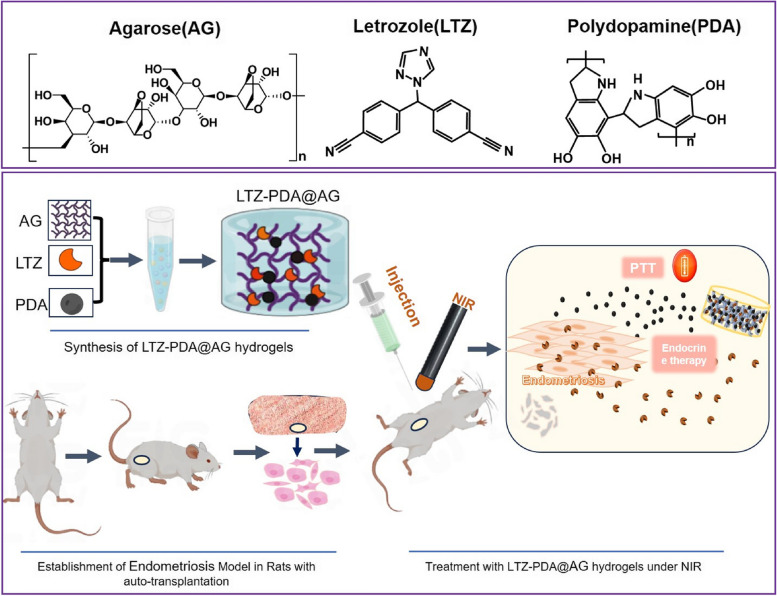
Synthesis of LTZ-PDA@AG hydrogels and endocrine therapy on endometriosis under NIR

In this study, we designed a hydrogel system that can be used for the treatment of endometriosis, namely LTZ-PDA@AG, a nanocomposite of polydopamine (PDA), LTZ, and AG hydrogels, which can be used to regulate the release of endocrine drugs under NIR radiation, as shown in Scheme 1. By regulating the AG content, the system can form a hydrogel to adhere to the lesion surface within the target time and further play a role in the follow-up mechanism and therapeutic effect. The composition of PDA in the hydrogel system has high biocompatibility and the ability to convert light energy into thermal energy, which leads to an increase in the temperature of the hydrogel matrix and the disintegration of the hydrogel matrix, thus releasing LTZ and decomposing it into oligomers synchronously, and then exiting the body with urine. Therefore, the LTZ-PDA@AG hydrogel delivery platform allows a non-toxic and biodegradable treatment of diseases and opens a new field of treatment for endometriosis.

## Materials and methods

### Ethical approval

This study was approved by the Ethics Committee of the Provincial Hospital of Shandong First Medical University. All patients enrolled in the trial signed an informed consent form to approve the use of their samples in the experimental study. The endometriosis group (*n* = 10) included patients with ovarian ‘chocolate cysts’ and eutopic endometrial (Eu) tissue. After harvesting, eutopic endometrial specimens were stored in phosphate-buffered saline (PBS) and placed in an ice box for primary cell extraction.

### Materials

Triblock poly (ethylene oxide)-b-poly (propylene oxide)-b-poly (ethylene oxide) Pluronic F127 (Mw 10,000–12000) was purchased from Energy Chemical Co., Ltd. (Shanghai, China). Dopamine hydrochloride (DA) was obtained from Macklin Biochemical Technology Co., Ltd. (Shanghai, China). Anhydrous ethanol and NH_3_·H_2_O were purchased from the Sinopharm Chemical Reagent Company (Shanghai, China). 1,3,5-Trimethylbenzene (TMB) and LTZ were purchased from Aladdin Reagent Co., Ltd. (Shanghai, China). AG was obtained from ABCONE Co., Ltd. (Shanghai, China). Calcein-AM and Propidium Iodide (PI) were purchased from Beyotime Biotechnology Co., Ltd. (Shanghai, China).

### Synthesis of LTZ-PDA@AG hydrogel

Based on a previous study by Liang et al. [42], PDA nanospheres with a photothermal effect were synthesized by simple stirring in a mixed system of water and ethanol. Briefly, Pluronic F127 (1 g) and DA (0.5 g) were dissolved in 100 mL of deionized water containing 50% anhydrous ethanol, and a clarified solution was obtained by magnetic stirring at room temperature at 600 rpm for 30 min. Subsequently, 2 mL of TMB was slowly injected into the solution, which was magnetic-stirred (500 rpm) at room temperature for 30 min. 5 mL of NH_3_·H_2_O was added dropwise to the above mixture to induce the self-polymerization of the dopamine oligomers, and the precise pH control was at a value of 8.5 which was strategically employed to govern the size modulation of the PDA nanoparticles. After continuous stirring for 30 min, the resulting product was centrifuged at 9000 rpm for 20 min. The resulting sediment was washed thrice with water and ethanol to obtain the PDA.

To prepare the LTZ-PDA@AG hydrogel, 62.5 mg of AG was dissolved in 20 mL of PBS containing 1 × TAE Buffer and the mixture was heated in a microwave to form an AG aqueous solution. When the solution cooled to 60 °C, 100 μL LTZ (2 mg/mL) and 100 μL PDA (100 mg/mL) were added with 30 s of stirring for uniform dispersion.

### Characterization

The microstructure of PDA was observed using a 120 kV transmission electron microscope (TEM, HT7800), and that of the PDA was observed using a scanning electron microscope (SEM). The hydrodynamic size of the PDA was determined using a Malvern Zetasizer (Nano-ZS, Malvern, U.K.). Fourier transform infrared (FTIR) spectra were obtained using an IRAffinity-1S spectrometer (Shimadzu, Japan). The UV–Vis absorption spectra of the PDA were measured using a Genesys 50 spectrophotometer.

### In vitro photothermal experiment

The photothermal effects of PDA and LTZ-PDA@AG were investigated at different power densities (1, 1.5, and 2 W·cm^−2^) using an 808 nm NIR. To evaluate the photothermal stability of the PDA, photothermal cycling experiments (five on/off lasers) were conducted. In each cycle, 808 nm laser with an irradiation power of 1.5 W·cm^−2^ was used to irradiate the PDA containing 1 mg/mL LTZ-PDA@AG hydrogel for 5 min, and then free cooled to room temperature without laser irradiation. A photothermal cycle test of the LTZ-PDA@AG hydrogel was performed in the same manner.

To determine the photo-controlled release behavior of LTZ in the LTZ-PDA@AG hydrogel in vitro, the UV–Vis absorbance of different concentrations of LTZ was detected using an ultraviolet spectrophotometer at 266 nm, and a LTZ standard curve was constructed according to its concentration and absorbance. The photothermal effect of the LTZ-PDA@AG hydrogel on the release rate of LTZ was studied using 808 nm NIR light. The concentration of released LTZ in the PBS solution was detected by UV–Vis spectrophotometry every 1 min. LTZ-PDA@AG hydrogel was incubated with 20 mL for 5 min without NIR, and then incubated for 5 min under 1.5 W·cm^−2^ with 808 nm NIR. The concentration of LTZ released from the supernatant at different time points was obtained from an LTZ standard curve.

The degradation behavior of the LTZ-PDA@AG hydrogel was evaluated based on its degradation rate (%). The LTZ-PDA@AG hydrogel was soaked in 20 mL of PBS. The experimental group was irradiated with 808 nm NIR for 5 min, and placed in a constant temperature shaker at 37 °C. The control group was placed in a constant-temperature shaker as well, and the hydrogel was removed and weighed every 12 h. The degradation rate was calculated as follows: Degradation rate (%) = (W_t_/W_0_) × 100 (W_t_ is the weight of the remaining hydrogel at different time intervals and W_0_ is the weight of the initial hydrogel).

To explore the dynamic volume change of LTZ-PDA@AG hydrogel, the expanded hydrogel disk with a thickness of 5 mm was irradiated with 808 nm NIR (1.5 W·cm^−2^) at 25 °C for 5 min and photographed every minute. The dynamic volume change rate was obtained according to the following formula: V_t_ /V_0_ = (d_t_ /d_0_)^3^; d_t_ and d_0_ are the hydrogel diameters at time *t* and at the initial time, respectively.

### In vitro therapy experiment

Endometrial tissues were harvested under sterile conditions from patients with endometriosis, washed with PBS, and homogenized. After centrifugation, three volumes of type I collagenase were added and the sample was digested in a 37 °C water bath for 40 min with vortexing every 10 min. Digestion was terminated by the addition of three volumes of DMEM/F-12 containing 10% fetal bovine serum to the sterile samples. The suspension was then filtered through 154 mm and 40 mm cell sieves. The cells were cultured in DMEM/F-12.

Human endometrial stromal cells (ESCs) were identified using immunofluorescence. Primary ESCs (1 × 10^5^/mL) were seeded onto coverslips in 24-well plates and incubated for 24 h. The coverslips were washed thrice with PBS and placed in 4% paraformaldehyde for 30 min. The cells were permeabilized in PBS containing 0.5% TritonX-100 at room temperature for 20 min, blocked with 0.5% bovine serum albumin (BSA) for 30 min, and then incubated with rabbit monoclonal antibodies against vimentin (1:100) and N-cadherin (1:200) at 4 °C overnight. The next day, the coverslips were washed and incubated with a fluorescent secondary antibody (1:200) for 1 h in a black wet box. DAPI was added to stain the nuclei for 10 min while avoiding exposure to light. The slides were mounted with gum-sealed tablets containing a fluorescence quenching inhibitor and imaged using a fluorescence microscope. The purity of the human endometrial stromal cells isolated in our study was over 95%.

Primary ESCs (1 × 10^6^/mL) were seeded in 6-well plates and incubated for 24 h, and divided into five groups: control, AG-LTZ, AG-LTZ-PDA, AG-LTZ-PDA + NIR, and NIR. The culture medium was replaced with fresh culture medium, and the hydrogels were placed at the center of the wells. After incubation for another 6 h, the cells were co-stained with Calcein AM and PI to detect the in vitro therapeutic effects. Another plate was stained with fluorescein isothiocyanate (FITC) and 7-amino-actinomycin D (7-AAD) and acoustic-focused flow cytometry (ATTUNE NXT) was used to analyze ESCs apoptosis.

### In vivo therapy experiment

The modeling principle is mostly based on the implantation theory of endometriosis for auto-transplantation. Female rats (*n* = 25; weight of ~ 200 g; 7 weeks) were purchased from Beijing Vital River Laboratory Animal Technology Co., Ltd. and housed in a specific pathogen‑free environment that was automatically maintained at a temperature of 23 ± 2 °C, a relative humidity of 45–65%, and with a controlled 12 h light/dark cycle. The animals had free access to food and water. The estrous cycle of the rats was confirmed using vaginal smears. Rats in estrus were anesthetized using Aver Din and fixed on an operating table. Iodophor was uniformly smeared over the abdomen, which was then cut open to locate the uterus. Two sides of the uterus were ligated along with the vessels using a twine, and one-centimeter of tissue was left in the middle. The 1-cm section of tissue was cut using a pair of scissors and placed in a sterile culture dish containing a normal saline solution. The endometrium was removed using sterile microscopic tweezers and cut into fragments measuring 5 × 5 mm. The fragments were attached to the abdominal walls. The incision was stitched and smeared with iodophor.

Following the induction of endometriosis, the rats were allowed to recover for four weeks, during which they were not administered any medication. Subsequently, 25 rats were divided into five groups: control (PBS), AG-LTZ, AG-LTZ-PDA, AG-LTZ-PDA + NIR, and NIR. The abdomen of each rat was cut open, and the corresponding treatments were administered via a one-time injection directly into the endometriotic implant (500 µL) following anesthesia with Aver din. The wounds were stitched and smeared with iodophor, and the animals were sacrificed eight days after treatment.

### Statistics analysis

Prism 7.0 (GraphPad, USA) and SPSS software (version 25.0; SPSS Inc., Chicago, IL, USA) were used for the statistical analyses. Student’s t-test and one-way ANOVA were used to compare data between the two groups. All data are reported as means ± the SD. *p* < 0.05 was considered statistically significant.

## Results

### Preparation and characterization of PDA and LTZ-PDA@AG hydrogels

**Fig. 1:**
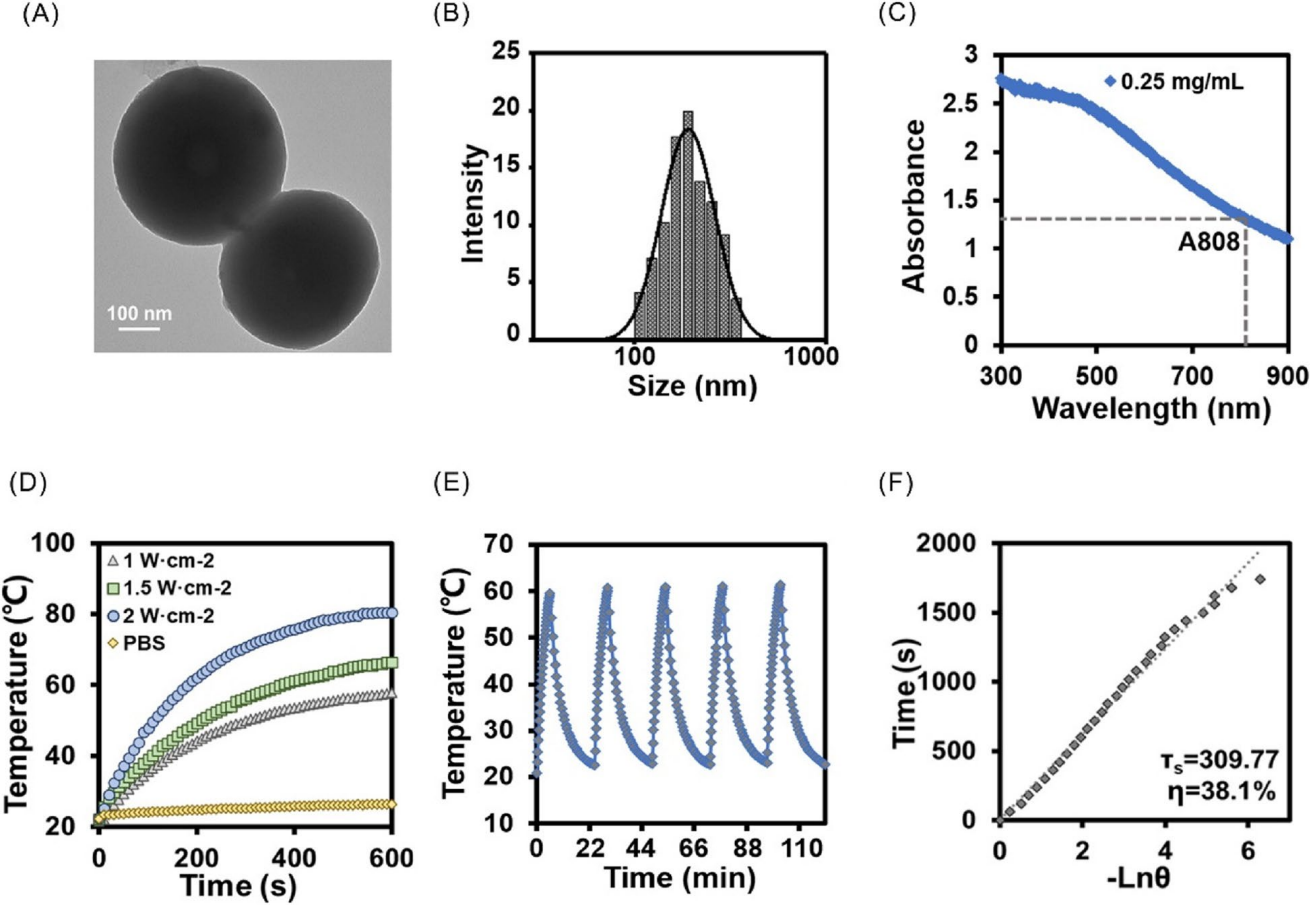
**A** TEM images of PDA. **B** The diameter of PDA measured by DLS analysis is in the range of 200–300 nm. **C** The UV–Vis absorbance of PDA. **D** The temperature changes of PDA solution at 808 nm NIR with different power. **E** The photothermal stability of PDA. Given NIR 5 min, then dropped to room temperature, repeated 5 cycles. **F** Schematic diagram of the negative natural logarithm relationship between temperature change and cooling time in the cooling phase of single-cycle photothermal stability test

The morphology of the PDA was characterized using TEM (Fig. 1A) and SEM (Figure S1). TEM images showed that PDA with a size of 200 nm had a uniform spherical structure. Dynamic light scattering (DLS) analysis showed that the hydrodynamic distribution of PDA was approximately 200–300 nm (Fig. 1B), which is close to the TEM results, proving that the PDA nanospheres had good dispersion. Absorption of nanomaterials in the NIR region is an important index of photothermal therapy (PTT). The UV–Vis absorbance of PDA (0.25 mg/mL) have a strong absorption at 808 nm (Fig. 1C). In addition, according to the Lambert–Beer's law, the extinction coefficient of PDA at 808 nm is 5.3 L ·g^−1^ cm^−1^ (Figure S2). To further explore the photothermal ability of PDA as a function of power, 808 nm NIR with different powers was used to conduct the photothermal experiment of the PDA solution (Fig. 1D). The results showed a power-dependent temperature enhancement, and the temperature increased nearly 25 °C when the power was 1.5 W·cm^−2^. These results indicate that PDA has good PTT potential. The photothermal effect of PDA did not deteriorate even when PDA underwent five heating processes (Fig. 1E), indicating that PDA nanomaterials have good photothermal stability and highlighting their potential as photothermal agents. The temperature record of a single photoperiod of PDA aqueous solution showed when the temperature rises to the highest (76 °C), the waiting temperature of NIR will be turned off to room temperature (Figure S3), and the photothermal conversion efficiency was calculated to be 38.1% (Fig. 1F). All those results indicated that PDA have a good photothermal conversion ability which is the basis for their application as photothermal agents in hydrogel systems.

**Fig. 2:**
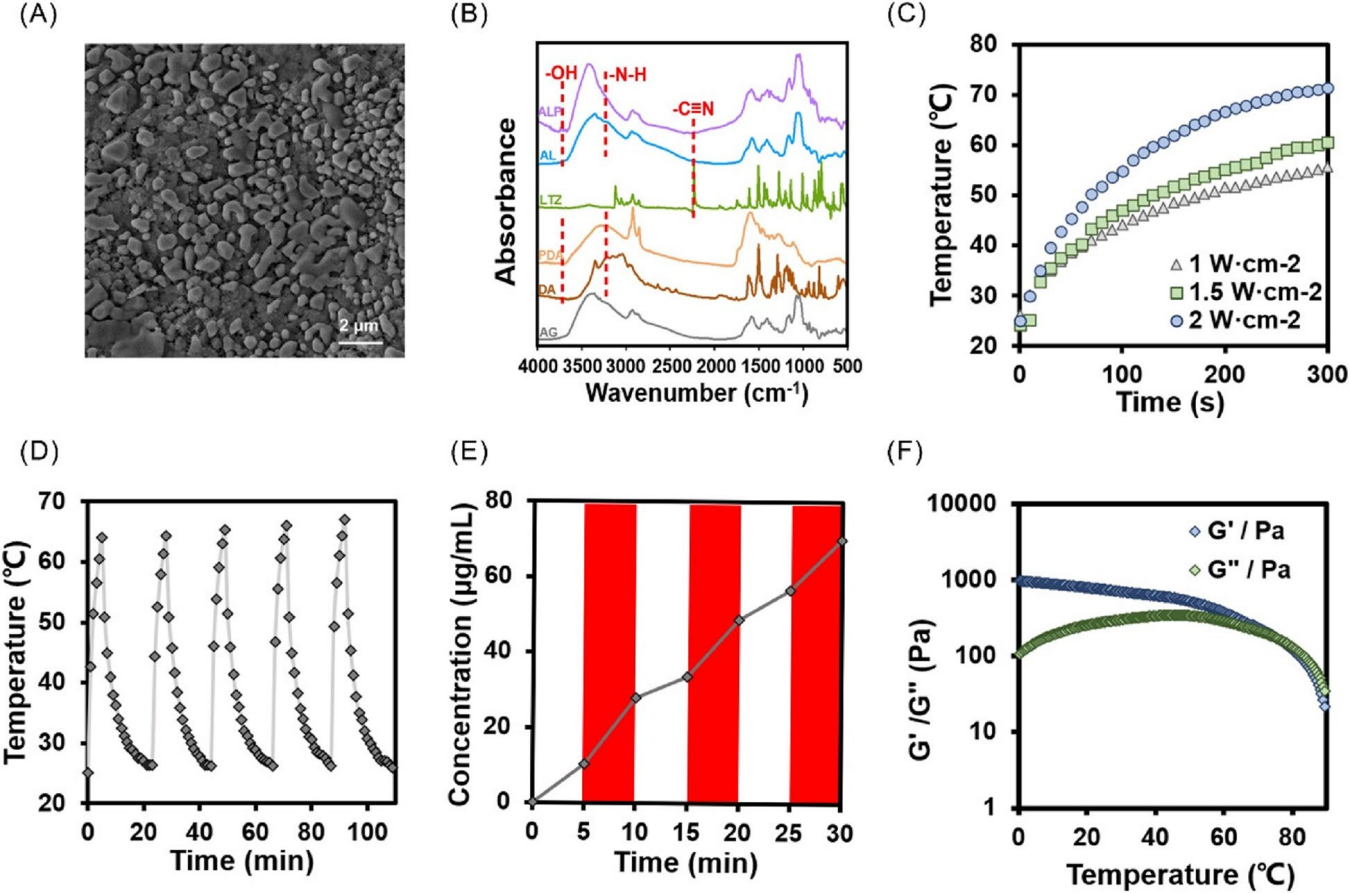
**A** Representative SEM images of LTZ-PDA@AG hydrogels. **B** Infrared measurements of each component (AG, DA, PDA, LTZ, ALN, ALPN) in LTZ-PDA@AG hydrogel in the wavelength range of 500 to 4000 cm^−1^. **C** Variation of hydrogel temperature with power after 808 nm NIR. **D** The photothermal stability experiment of LTZ-PDA@AG hydrogels with 1.5W cm^−2^ NIR irradiation power. **E** Accumulated release of LTZ from LTZ-PDA@AG hydrogels with and without 808 nm NIR. **F** The changes of storage modulus (G') and loss modulus(G'') at different temperatures

As shown in Fig. 2A, the SEM images show that LTZ and PDA were successfully compounded on the hydrogel surface. To verify whether LTZ and PDA were successfully loaded into the hydrogel, FTIR spectra of ALPN was measured. The results showed that the absorption peak at 2240 cm^−1^ can be attributed to -C≡N stretching vibration, and those at 3250 cm^−1^ and 3600 cm^−1^ can be attributed to the -NH_2_ and-OH stretching vibration, respectively, which confirms that LTZ and PDA were successfully encapsulated in AG hydrogel carrier (Fig. 2B). Next, the photothermal properties of LTZ-PDA@AG hydrogel were detected. The LTZ-PDA@AG hydrogel containing 1 mg/mL PDA was irradiated with different power (1, 1.5, 2 W cm^−2^) of 808 nm NIR (Fig. 2C). When the power was 1.5 W·cm^−2^, the LTZ-PDA@AG hydrogel was heated to 60 °C in 5 min, which fully met the requirements of LTZ photothermal controlled release. At the same time, the photothermal stability of LTZ-PDA@AG hydrogel was measured when the NIR irradiation power was 1.5 W·cm^−2^(Fig. 2D). The results show that the photothermal effect of the LTZ-PDA@AG hydrogel did not deteriorate during heating, indicating that the designed hydrogel system had good thermal stability and achieved a stable and continuous photothermal effect. Within the hydrogel drug-loading system we have conceptualized, agarose, acknowledged for its thermos-responsive characteristics, undergoes a physical reaction induced by the photothermal conversion of polydopamine (PDA) under Near-Infrared (NIR) irradiation. This transformation triggers the skeleton structure breakdown and disintegration of the hydrogel framework into smaller molecular entities, thereby fostering an environment conducive to physiological degradation. According to the standard curve of LTZ (Figure S4), Fig. 2E shows the release of LTZ from the LTZ-PDA@AG hydrogel caused by the increase in light-controlled temperature, which was approximately twice as much as that when light was turned off. The outcome of this meticulous examination revealed an absence of discernible alteration in the cytotoxic effect of letrozole on ESCs within the realm of elevated thermal conditions. Because the chemical structural stability of the compounds is closely related to the corresponding UV–vis absorption, we have conducted a meticulous analysis of its UV–vis absorption of LTZ against heat or pH. The comprehensive scrutiny of LTZ's absorbance profiles across a spectrum of temperatures and pH levels yielded no statistically significant variations. This observation strongly supports the assertion that the fundamental chemical structure of LTZ is stable within these conditions (Figure S5). Then rheological tests were performed on the LTZ-PDA@AG hydrogels to analyze their mechanisms. The ratio of G'' to G' was expressed by tan δ (Figure S6). The changes in the storage modulus (G') and loss modulus (G’) of the LTZ-PDA@AG hydrogels at different temperatures were recorded (Fig. 2F). The results showed that the storage modulus and loss modulus decreased rapidly with the increase of temperature, and δ increased rapidly after 65 °C. This suggests that the material changes from a gel state to a liquid state, indicating that temperature has a significant influence on the viscosity of the LTZ-PDA@AG hydrogel. The above result indicates that the concentration of LTZ increased sharply compared with that without NIR irradiation, indicating that the temperature rise of LTZ-PDA@AG was flexibly regulated by external NIR light, thus realizing the controlled release of LTZ.

**Fig. 3:**
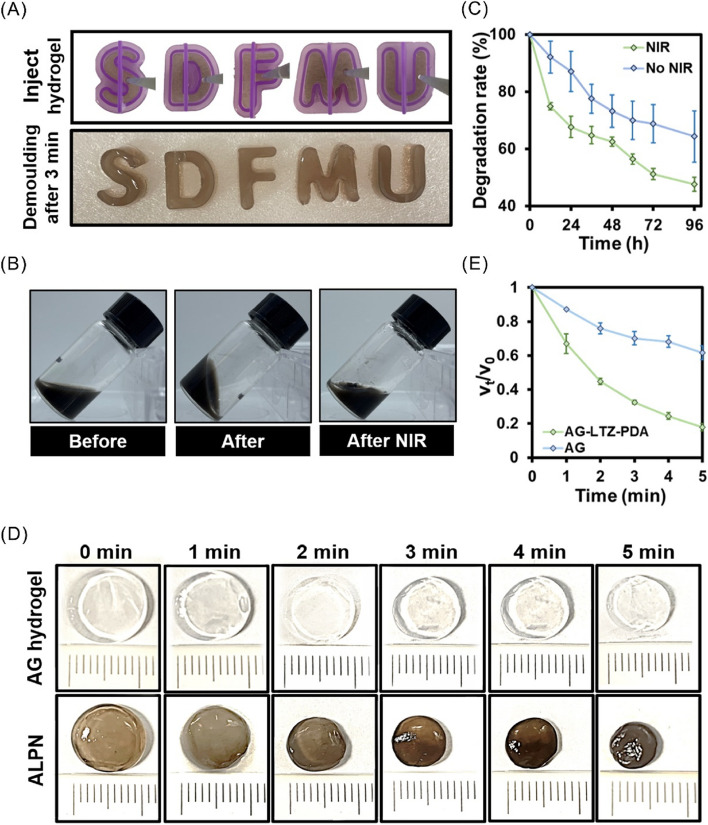
**A** Injectable mobile hydrogel, and photographs of the gel's writing by injection. **B** Photos of hydrogels before and after gelation, and after NIR. **C** The degradation degree of LTZ-PDA@AG hydrogels with or without NIR. **D** The images of contraction response ability of LTZ-PDA@AG hydrogels with or without NIR and (**E**) The corresponding quantitative analysis

After the liquid gel injected into the mold for 3 min, and the letter "SDFMU" is fully displayed in the original position (Fig. 3A). When the hydrogel in a flow state was injected into the bottle, the liquid gathered at the bottom of the unlabeled side, and the hydrogel still gathered on the unlabeled side after converting the bottle at 180°, indicating that the hydrogel system had good gel-forming properties. After NIR irradiation for 5 min, the hydrogel became semi-flowing and reflowed to the bottom of the unlabeled side (Fig. 3B). These results indicated that the hydrogel had high plasticity and agglutination properties. The degradability of the LTZ-PDA@AG hydrogels can improve their clinical application potential. To further evaluate the NIR photodegradation ability of the LTZ-PDA@AG hydrogel, we measured its quality to obtain different degrees of degradation with and without NIR irradiation (Fig. 3C). The results showed that the degradation rate of the LTZ-PDA@AG hydrogel irradiated with NIR was approximately 20% higher than that of the group without NIR irradiation, indicating that the LTZ-PDA@AG hydrogel had a strong NIR photodegradation ability. The LTZ-PDA@AG hydrogel attains a degradation extent of 50% within 96 h. To comprehensively elucidate this process, supplementary investigations were conducted, resulting in the observation of near-complete degradation of the LTZ-PDA@AG hydrogel at the 16-day mark (Figure S7). Furthermore, strategic augmentation of individual component concentrations within the system demonstrates a prospective avenue to extending the release duration to a certain extent. Compared with the AG hydrogel, the LTZ-PDA@AG hydrogel showed a rapid contraction-response behavior when irradiated with NIR (Fig. 3D and E). These results indicate that the LTZ-PDA@AG hydrogel can control the release and degradation of NIR, which is of great value in the clinical application of endometriosis.

### In vitro PTT of endometriosis

**Fig. 4:**
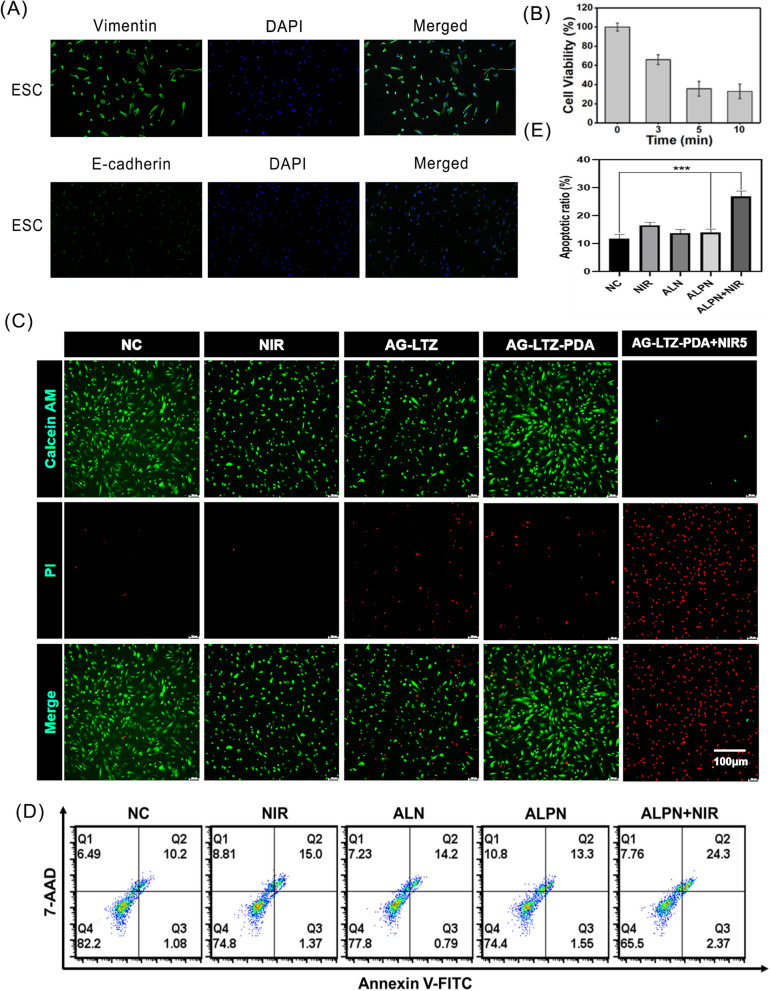
In vitro photothermal therapy of LTZ-PDA@AG hydrogel. **A** Discriminant analysis of ESCs. **B** The Relationship between PTT Ability and NIR time. **C** Confocal images for live and dead staining analysis with different treatments. **D**, **E** Flow cytometry analysis of apoptosis rate of endometriosis cells with different treatments

ESCs were extracted from the endometrial tissues and identified as shown in Fig. 4A, indicating that the purity of ESCs was high enough for follow-up experiments. To explore the photothermal ability of NIR radiation and the biocompatibility for other types of cells, MTT experiments were performed to detect the effects of PTT and cells viability. The empirical findings substantiate that the viability of 3T3 and HUVEC cells, following exposure to the hydrogel, exceeded 80% (Figure S8). This outcome serves as demonstrative evidence underscoring the commendable biocompatibility extended to diverse cell types beyond the scope of investigation. As shown in Fig. 4B, the cell survival rates were 67% at 3 min and 38% at 5 min. PDA decomposed gradually under NIR irradiation and almost completely decomposed in 5 min, indicating that its photothermal effect was greatly reduced, even though the delay of light time did not change the cell survival rate. Therefore, 5 min was selected as the optimal exposure time. To further verify the effect of PTT, the results of Calcein AM/PI staining showed that the NIR group had no red fluorescence owing to the lack of photothermal material, which was in accordance with the NC group. Because of the absence of NIR light, only a small amount of LTZ was released in the AG-LTZ and AG-LTZ-PDA groups. Red fluorescence was slightly stronger than that in the previous two groups; however, there was no significant difference between them. The ALPN group had the strongest red fluorescence compared to the other groups, indicating a significant cell-killing effect under NIR irradiation (Fig. 4C). This phenomenon was further validated by classical flow cytometry, which confirmed that apoptosis induced by PTT caused a large number of endometriotic cell deaths (Fig. 4D). In the same inflammatory environment of atherosclerotic disease, the CuS/TiO_2_ heterostructured nanosheets could induce up to 90% apoptosis of macrophages under NIR irradiation [43], and single-walled carbon nanotubes were applied in promoting the apoptosis of macrophages as photothermal conversion agents [44]. Similarly, Peng et al. performed polypyrrole nanoparticles mediated NIR photothermal treatment on macrophages causing arterial restenosis which exists chronic inflammation and abnormal cell proliferation, showing that the treated group had a significantly higher apoptotic index [45]. Additionally, Marghani et al. used silver nanoparticles as photothermal conversion agents in PTT of benign prostatic hyperplasia disease, which induced apoptosis apparently [46]. Dong et al. considered that PTT can convert light into heat under light irradiation, or generate ROS, to promote necrosis or apoptosis of rheumatoid arthritis inflammatory cells [47]. Pourhajibaghe et al. demonstrated that apoptosis caused by PTT led to human gingival fibroblast cells death by verifying increased expression of apoptosis-related BAX messenger RNA levels [48]. Like above non-tumor cells, endometriosis cells could send out signals after receiving mild photothermal stimulation for a short period of time, causing a series of events such as intracellular heat stress, mitochondrial damage, oxidative stress, DNA damage and caspase activation, thereby triggering cell apoptosis in response to environmental changes. All the above results demonstrate the potential application of near-infrared light-controlled release hydrogel combinations in the treatment of endometriosis.

### In vivo PTT of endometriosis

**Fig. 5:**
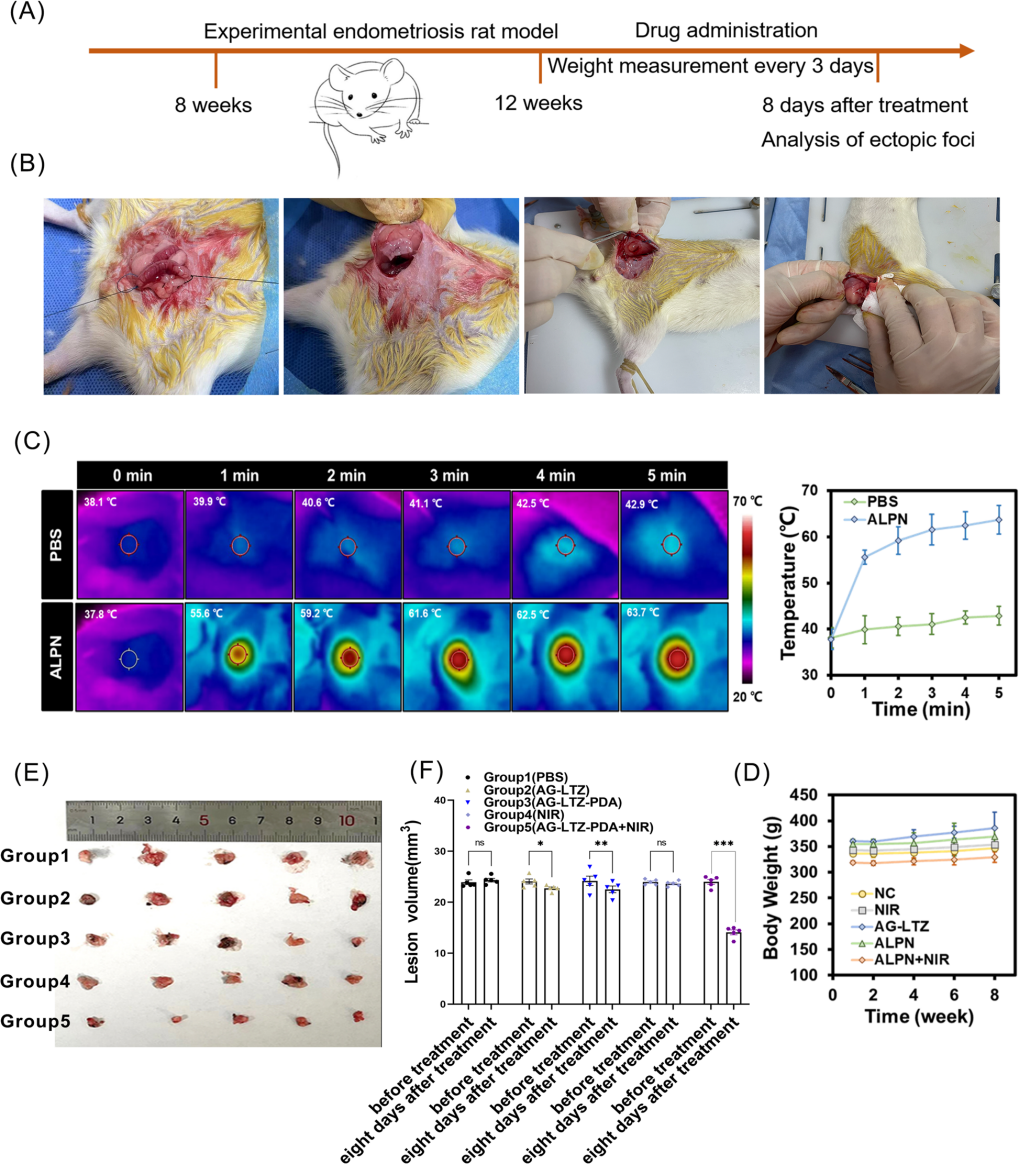
**A** Schematic illustration of treatment process in rats. **B** The model of endometriosis. **C** In vivo photothermal effects of Hydrogel platform. **D** The body weights of rats before and after treatment. **E**, **F** The volume of foci before and after treatment

**Fig. 6:**
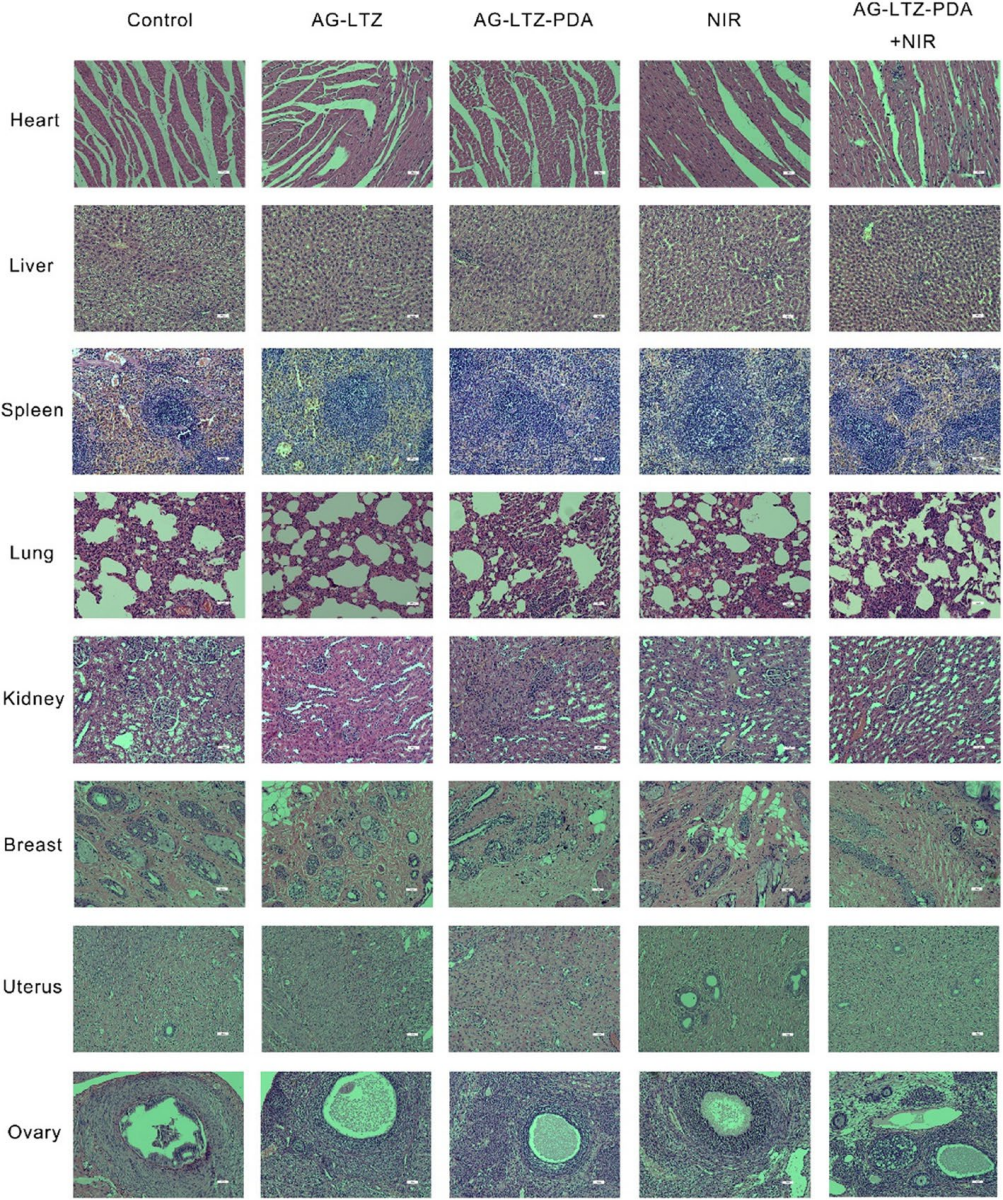
H&E staining of main organs (heart, liver, spleen, lung, kidney, breast, uterus, and ovary) from rats after treatment. Scale bar = 100 μm

Based on the biological effects described above, an in vivo experiment was performed to validate the therapeutic effect on endometriosis; the scheme is shown in Fig. 5A. The most used model of endometriosis is rat auto-transplantation, and we successfully established a model of endometriosis (Fig. 5B). Four weeks after auto-transplantation, we observed an endometrial lesion in the hypogastrium of every rat, and there was no significant difference in lesion volume. The rats were then randomly divided into five groups and administered the corresponding treatments. After injection of the hydrogel, animal photothermal images were monitored using a thermal camera after 5 min of NIR irradiation. Compared with free LTZ group (only 5 °C increase), the local foci temperature of the ALPN group rapidly rose to 63.7 °C, which revealed its good photothermal therapeutic potential (Fig. 5C). In line with the volume of foci before and after treatment shown in Fig. 5E and F, the foci volume of the first four groups showed no significant differences; however, the AG-LTZ-PDA + NIR group showed obvious differences. Compared to the AG-LTZ, AG-LTZ-PDA, and NIR groups, the volume of endometriotic foci was significantly reduced in the AG-LTZ-PDA + NIR group. This result indicates that AG-LTZ-PDA could successfully play a role in the in vivo endocrine therapy of endometriosis through PTT. Because the properties of the hydrogel were proven after eight days of treatment in vivo, to further explore its biosafety and biocompatibility, major organs of the mice were sliced and stained with hematoxylin and eosin (H&E) for histological analysis. As shown in Fig. 6, rats that were killed eight days after AG-LTZ-PDA injection with NIR irradiation exhibited no significant damage to normal tissues, including the heart, liver, spleen, lung, kidney, breast, uterus, and ovary. No weight loss was observed in any treatment group during this period (Fig. 5D). Collectively, these results confirmed that AG-LTZ-PDA + NIR treatment had no observable side effects or toxicity to normal tissues and is promising for potential clinical applications in endometriosis therapy.

## Discussion

Currently, medical treatments for endometriosis aim to reduce and eliminate the focus, relieve, and eliminate pain, reduce, and avoid recurrence, and improve and promote fertility. Medroxyprogesterone acetate, danazol, oral contraceptives, and GnRH-a are effective in alleviating pain-associated symptoms of endometriosis and the regression of endometriotic lesions. Aromatase p-450 is the key enzyme in estrogen biosynthesis, as it catalyzes the conversion of androstenedione and testosterone to estrone and estradiol. LTZ is an aromatase inhibitor that competitively binds to the heme group of the cytochrome P450 subunit, thereby reducing estrogen levels. However, adverse effects, such as nausea, pain, and hot flashes, limit their long-term use and high recurrence rates after the cessation of therapy. Therefore, new agents that present synchronous fertility treatments with improved side effect profiles are needed.

LTZ-PDA@AG is an intelligent drug-release platform with an NIR response constructed by adjusting the proportion of various components in the hydrogel. The solid-state LTZ-PDA@AG transformed into a gel state after injection into the endometriotic foci because of the lower body temperature, resulting in a phase transition. The AG content was AG to form a hydrogel that adhered to the surface of the lesion within the target time. PDA has high biocompatibility and the ability to convert light energy into thermal energy, which leads to an increase in the hydrogel matrix temperature and disintegration of the hydrogel matrix, releasing LTZ, and synchronously degrading it into oligomers. The in vitro and in vivo experiments showed that LTZ-PDA@AG killed ESCs and reduced the number of endometrial foci. Notably, LTZ-PDA@AG showed negligible toxicity towards ESCs, and both AG hydrogels were degradable when the treatment was completed.

Medicine and surgery are the primary treatments for endometriosis. Medical therapy should be conceived as a long-term treatment, similar to the therapy for other chronic inflammatory conditions. Symptom recurrence after drug discontinuation was expected and did not demonstrate manipulation inefficacy. In addition, some drugs create a hypoestrogenic (GnRH agonist), hyperandrogenic (danazol, gestrinone), or hyper-progestogenic (oral contraceptives, progestins) environment with the suppression of endometrial cell proliferation. Pharmacological treatments are symptomatic and not cytoreductive; lesions survive any drug, at any dose, for any period of use, and are ready to resume their metabolic activity at treatment discontinuation. Therefore, it is extremely important to effectively reduce the number of foci and delay recurrence. Currently, there exists a substantial body of literature documenting the utilization of photothermal-controlled release hydrogel systems in various fields, including cancer treatment and wound healing. However, it is noteworthy that this is the first time to propose the application of a photothermal controlled-release hydrogel system in the treatment of endometriosis. It is important to highlight that most hydrogel carriers employed in previous studies exhibit a singular carrier function and possess complex compositions. Examples include the utilization of PNIPAM polymers [49], alginate solutions [50], PLGA-PEG-PLGA triblock copolymers [51], and analogous materials. In contrast, the hydrogel system employed in our study incorporates agarose gel, serving not only as a drug carrier but also as a straightforward temperature-responsive hydrogel. Significantly, it is essential to emphasize that agarose has received safety certification from the U.S. Food and Drug Administration (FDA) [28]. Moreover, our methodology involves the incorporation of PDA and LTZ within the temperature range of 40 °C to 50 °C, a decision guided by multiple considerations. On one hand, it considers the heat sensitivity of PDA, which is susceptible to decomposition under thermal stress [52]. On the other hand, this approach preserves the pharmaceutical activity of LTZ. During the in vivo experiment, the state of the hydrogel was consistent with that in the in vitro experiment, and the treatment mode, such as illumination time, dosage, etc., was also completely consistent. Therefore, the duration and speed of drug release in the body can be completely referred to the in vitro experiment. In the context of endometriosis treatment, this dual-modality strategy allows for the simultaneous implementation of both treatment approaches, potentially resulting in a level of efficacy surpassing conventional methods. The photoconverting capability of PDA imparts controlled release functionality to the hydrogel system, facilitating the continuous administration of LTZ for endocrine therapy while mitigating the risk of adverse reactions.

A majority of endometriosis lesions are predominantly localized on the surfaces of pelvic and abdominal organs in female patients. The incorporation of therapeutic agents within the hydrogel matrix enables precise and controlled drug release, facilitating targeted drug delivery to the intended site. This attribute proves especially invaluable when addressing deeply entrenched endometriotic lesions within the abdominal cavity, where conventional treatment modalities encounter inherent challenges. While it's important to note that the LTZ-PDA@AG system may not provide complete abdominal cavity coverage, it can be skillfully introduced via laparoscopic methods or guided by ultrasound to target the lesions, ensuring extensive distribution around the lesions, and optimizing therapeutic efficacy. As a result, this approach holds great promise within the clinical application landscape. Additionally, the effectiveness of the LTZ-PDA@AG system in treating endometriotic lesions has been rigorously validated through both in vivo and in vitro experiments. These findings underscore its considerable potential for translation into clinical diagnostics and therapeutics, reaffirming its significance.

However, limitations inherent within our research framework must be acknowledged. Firstly, the occult nature of lesions located in anatomically distant regions or of small size hinders early detection and thus evades timely therapeutic intervention. Secondly, even LTZ-PDA@AG hydrogel achieved complete coverage of all ectopic lesions, due to the limited irradiation region, NIR can only act on a part of endometriosis lesion one time, causing signal response of endometriosis cells and induce their apoptosis. Thirdly, our current hydrogel systems lack ligand-targeted therapy for endometriosis cells. Therefore, relevant immunotherapy and ligand-targeted therapy can be added in subsequent studies, or more suitable drug delivery systems can be explored. This consideration is consistent with the ongoing pursuit of refining treatment strategies to achieve optimal efficacy while ensuring patient safety.

## Conclusion

We developed an injectable LTZ-PDA@AG hydrogel as an implant for synergistic local photothermal endometriosis therapy. The LTZ-PDA@AG hydrogel exhibited gelation characteristics and excellent NIR-responsive properties, requiring only 3 min to form a gel from the liquid state. After 5 min NIR irradiation, the local foci temperature of the ALPN group rapidly rose to 63.7 °C, while temperature of the free LTZ group only increased 5 °C. As inferred from the LTZ release curve, the amount of LTZ released from the LTZ-PDA@AG hydrogel caused by the increase in the NIR-controlled temperature was approximately twice as much as that without NIR. By comparing the changes in lesion volume before and after the treatment, the foci in the AG-LTZ-PDA + NIR group were significantly reduced compared to those in the other groups. The results showed that NIR irradiation accelerated the release of LTZ and improved its therapeutic effect. Additionally, biosafety and biocompatibility analyses showed that the LTZ-PDA@AG hydrogel had hardly any toxic effects on the main organs by H&E staining and weight detection in experimental rats. Therefore, this NIR-responsive LTZ-PDA@AG hydrogel is a promising candidate for synergistic endometriosis therapy.

### Supplementary Information

**Additional file 1:** **Figure S1.** SEM images of PDA. **Figure S2.** The extinction coefficient of PDA at 808 nm. **Figure S3.** The temperature record of a single photoperiod of PDA solution. **Figure S4.** The standard curve of LTZ. **Figure S5.** UV-vis spectra of LTZ under different temperature or pH condition. **Figure S6.** The ratio of G'' to G' was expressed by tan δ.**Figure S7.** The degradation curve of LTZ. **Figure S8.**The biocompatibility of 3T3 and HUVEC cells.

## Data Availability

The datasets used and analyzed during the current study are available from the corresponding author on reasonable request.

## Abbreviations

LTZ: Letrozole
PDA: Polydopamine
AG: Agarose
PTT: Photothermal therapy
TMDs: Transition metal disulfides
PTAs: Photothermal energy exchangers
NIR: Near-infrared
FDA: Food and Drug Administration
Eu: Eutopic endometrial
PBS: Phosphate-buffered saline
DA: Dopamine hydrochloride
TMB: 1,3,5-Trimethylbenzene
PI: Propidium Iodide
TEM: Transmission electron microscope
SEM: Scanning electron microscope
FTIR: Scanning electron microscope
ESCs: Endometrial stromal cells
FITC: Fluorescein isothiocyanate
7-AAD: 7-Amino-actinomycin D
DLS: Dynamic light scattering
H&E: Hematoxylin and eosin
