# Supplementary material for: Injectable hydrogel nanoarchitectonics with near-infrared controlled drug delivery for in situ photothermal/endocrine synergistic endometriosis therapy
Source: Biomater Res. 2023 Oct 7;27:100. doi: 10.1186/s40824-023-00442-2 (PMC10560439; doi:10.1186/s40824-023-00442-2)
Supplement: Supplementary file 1 — Additional file 1: Figure S1. SEM images of PDA. Figure S2. The extinction coefficient of PDA at 808 nm. Figure S3. The temperature record of a single photoperiod of PDA solution. Figure S4. The standard curve of LTZ. Figure S5. UV-vis spectra of LTZ under different temperature or pH condition. Figure S6. The ratio of G'' to G' was expressed by tan δ.Figure S7. The degradation curve of LTZ. Figure S8.The biocompatibility of 3T3 and HUVEC cells. [file 40824_2023_442_MOESM1_ESM.docx]

**Injectable Hydrogel Nanoarchitectonics with Near-Infrared Controlled Drug Delivery for In Situ Photothermal/Endocrine Synergistic Endometriosis Therapy**

Wei Tian^1, #^, Chenyu Wang^1, #^, Ran Chu^1^, Haiyan Ge^2^, Xiao Sun^2,^ *, Mingjiang Li^1,^ *

^1^Department of Gynecology, Shandong Provincial Hospital Affiliated to Shandong First Medical University, Jinan, Shandong, China.

^2^School of Chemistry and Pharmaceutical Engineering, Medical Science and Technology Innovation Center, Shandong First Medical University, Jinan, China.

^#^These authors contributed equally to this work.

*Correspondence:

E-mail address: limingjiang1963@126.com (M. Li); sunxiao@sdfmu.edu.cn (X. Sun)

**Supplementary**


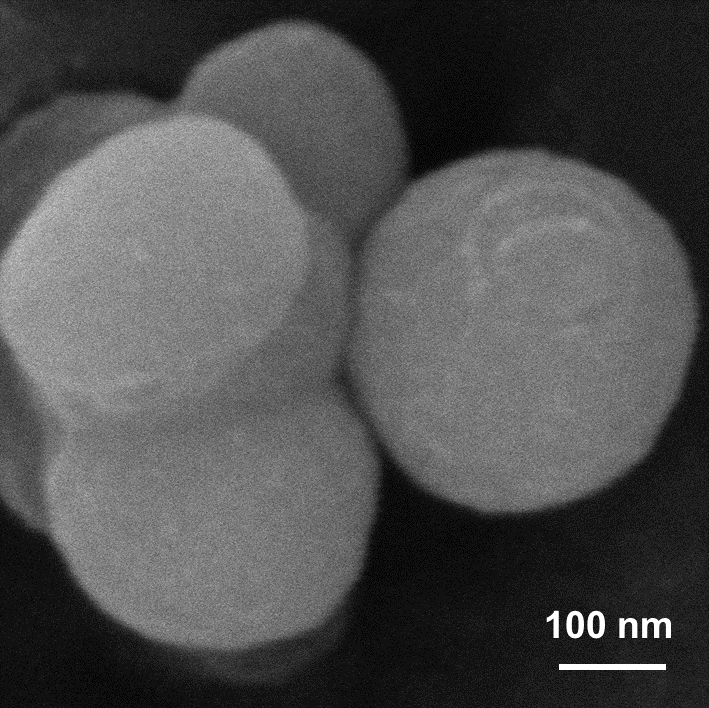


Figure S1. SEM images of PDA.


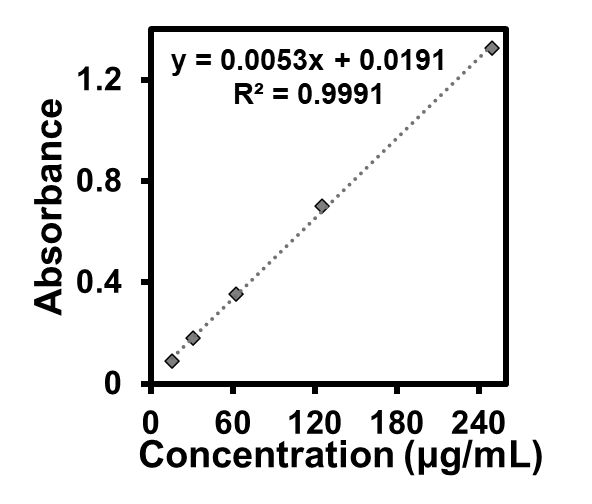


Figure S2. The extinction coefficient of PDA at 808 nm.


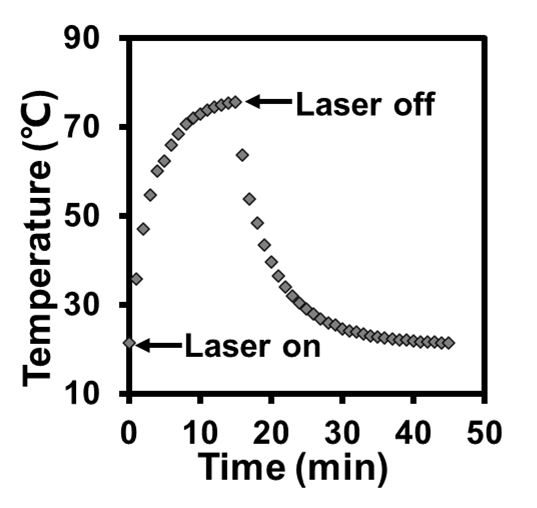


Figure S3. The temperature record of a single photoperiod of PDA solution.


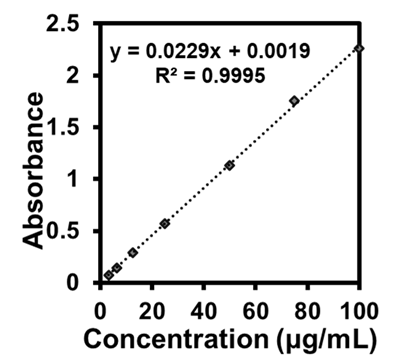


Figure S4. The standard curve of LTZ.


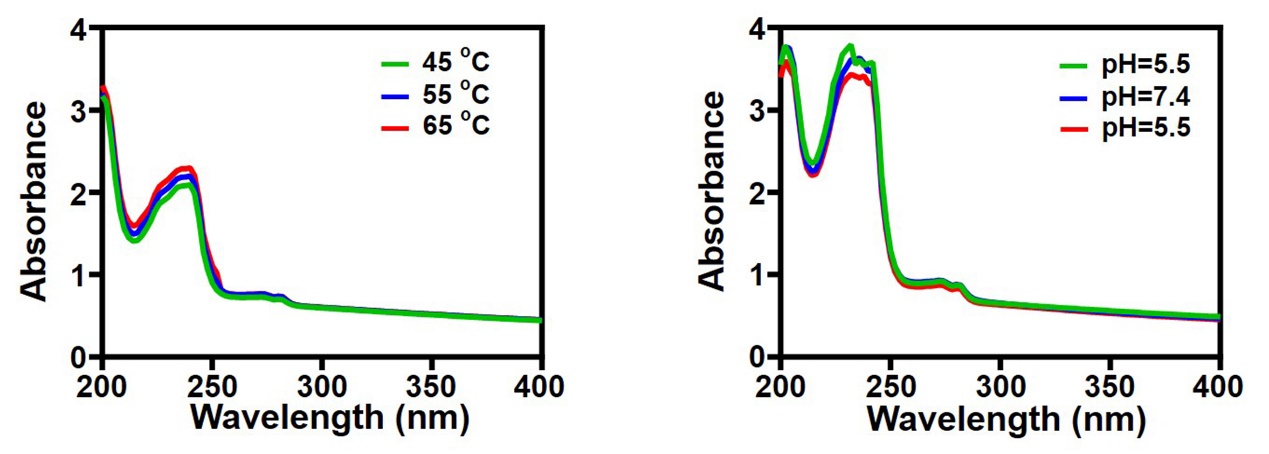


Figure S5. UV-vis spectra of LTZ under different temperature or pH condition.


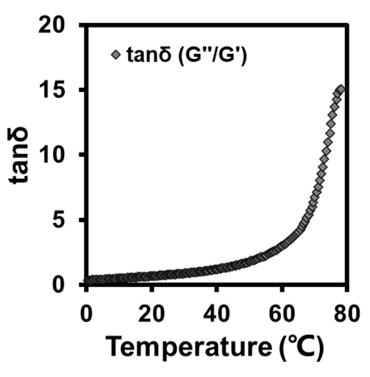


Figure S6. The ratio of G'' to G' was expressed by tan δ.

Figure S7. The degradation curve of LTZ.


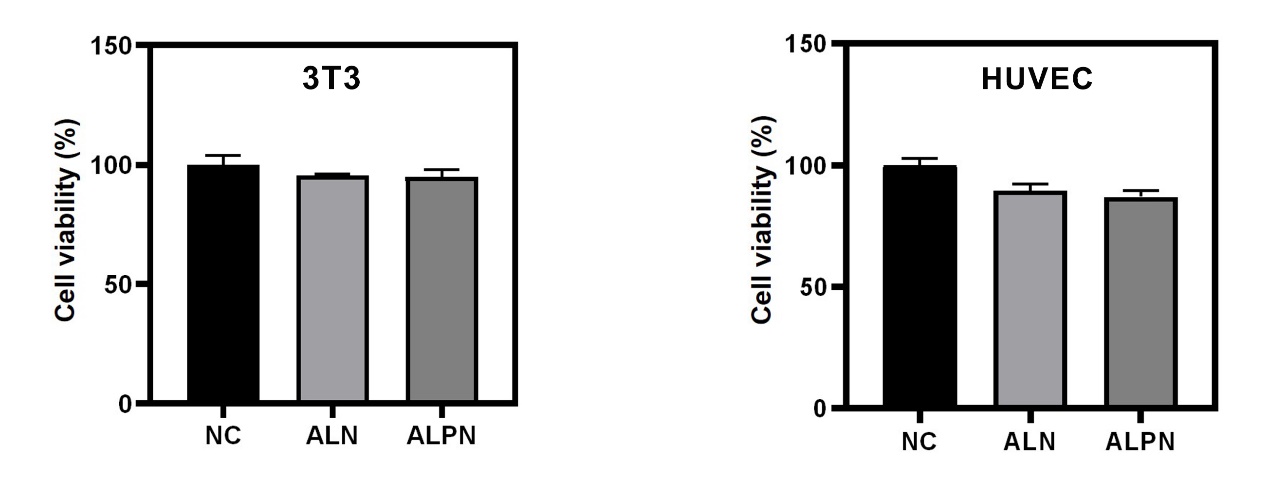


Figure S8. The biocompatibility of 3T3 and HUVEC cells.
